# Expectations of citizens from the government in response to COVID-19 pandemic: a cross-sectional study in Iran

**DOI:** 10.1186/s12889-021-10722-y

**Published:** 2021-04-08

**Authors:** Leila Zarei, Saeed Shahabi, Ahmad Kalateh Sadati, Reza Tabrizi, Seyed Taghi Heydari, Kamran Bagheri Lankarani

**Affiliations:** 1 Health Policy Research Center, Institute of Health, Shiraz University of Medical Sciences, Shiraz, Fars Iran; 2 Department of Social Sciences, Yazd University, PO Box: 98195–741, University Blvd, Safayieh, Yazd, Iran

**Keywords:** Expectations of citizens, The response of government, COVID-19, Pandemic, Cross-sectional survey, Iran, Transparency, Burden

## Abstract

**Background:**

The government is the main body in charge of controlling epidemics; hence, expectations from the intention and capacities of the government would affect the flexibility and behaviors of citizens. Given the severity of COVID-19 pandemic and the urgent need for cooperation of people in the prevention and combat processes, understanding the public perspectives would be crucial and instructive. This study aimed to explore such perspectives towards the current pandemic among the Iranian. Indeed, we sought to provide a favorable platform for effective policies in the face of the COVID-19 pandemic through recognizing public expectations.

**Methods:**

This cross-sectional survey used an open-ended online questionnaire to investigate the common perspectives of the Iranian towards the response of government to COVID-19 pandemic. The participants were selected using snowball and convenient sampling techniques across the country. The collected data were analyzed and described using a thematic analysis.

**Results:**

In general, 2547 participants agreed to participate in this study and completed the online questionnaire. According to the findings, the Iranian exhibited several expectations regarding the response of the government to COVID-19 pandemic. Three main themes were extracted based on these expectations: (1) health-related expectations, (2) policy-related expectations, and (3) Information-related expectations. In this study, a majority of participants highlighted the need to consider and follow-up the patients and their families, providing the financial and hygiene support during the pandemic, applying strict restrictions, and using close monitoring and controlling procedures. Furthermore, they mentioned that authorities and news agencies should observe the principals honesty and transparency.

**Conclusions:**

Our findings revealed that people expect the government and other responsible institutions to minimize the burden of this pandemic through adopting effective policies. Also, they could help policy-makers become aware of the expectations of people and develop better strategies.

**Supplementary Information:**

The online version contains supplementary material available at 10.1186/s12889-021-10722-y.

## Background

After numerous reports on an unknown respiratory disease in Wuhan, China, at the end of 2019, a novel coronavirus, which is known as Severe Acute Respiratory Syndrome Coronavirus 2 (SARS-CoV-2), was detected as the main cause of the disease (COVID-19) [1]. Almost three-million positive cases and more than 207,000 deaths have been recognized globally since April 26 [2]. Following this pandemic, countries applied different policies such as social distancing and lockdown measures to control it [3]. Due to the fact that there is no specific treatment strategy for COVID-19, the best technique to fight this outbreak is to follow the personal hygiene and preventive measures along with active case detection and isolation in order to reduce the possibility of virus transmission [4].

Following the detection of two COVID-19 cases on February 20 in Iran, this pandemic spread rapidly across the country, and more than 90,000 positive cases and about 5700 deaths were reported by April 26 [2]. Consequently, several strategies and policies were developed by the government of Iran in order to control this outbreak including social distancing, and prohibition of unnecessary trips and closure of educational centers [5]. Although applying the above-mentioned approaches had positive effects on the pandemic control [6], they led to serious challenges for the society, especially for the poor and vulnerable groups. Due to the imposition of international sanctions on Iran throughout the last decades, the negative effects of this pandemic on the already-weakened economy were enormous [7].

Evidence suggests that epidemics such as COVID-19 have significant economic and social consequences such as unemployment, poverty, and stigmatization [8]. The United Nations (UN) and other international organizations have predicted that the negative economic impacts of this pandemic are harsh, especially for developing countries [9, 10]. The enhancement of the domestic violence is considered as another consequence of COVID-19 outbreak [11]. Following the containment measures, a 30% increase in domestic violence has been reported in Europe [12].

Sociologically, the policy effectiveness and governance agenda in crisis are the main expectations of citizens in this modern public sphere. Protecting the lives of citizens is one of the first duties of the governments that can also shape the public expectations of citizens. Such expectations seem to affect the behaviors and attitudes of citizens toward public services [13]. In public health emergencies arising from an infectious disease epidemic, the government is in charge of protecting the community residents by preventing the spread of the disease [14]. The expectations of citizens from the capabilities and policies of the government can influence their decisions [15]. The government is also responsible for regulating and controlling health activities, sanitation services, and epidemiologic surveillance [16].

In a previous study, the social concerns related to the COVID-19 pandemic were investigated from different perspectives. For example, Nelson et al. (2020) had assessed public concerns about the COVID-19 crisis in the United States before shelter-in-place orders were widely implemented [17]. A new study of public attitudes across Europe, America and Asia has found that people in the UK have the highest levels of concern about coronavirus – more than Italy or Spain – while those in South Korea are the least concerned [18]. Stokes et al. (2020) claimed that public response to the pandemic is important; however, they are being measured and it could lead to an earlier recognition of changing public priorities, fluctuations in wellness, and uptake of public health measures. All of the mentioned factors could have implications for individual- and population-level health [19].

The formulation and adoption of effective, equitable, and community-based policies must be considered to diminish the burdens of such epidemics [20]. The community-based policies are on the basis of community engagement, and play a vital role in controlling COVID-19; therefore, they have to be considered in the processes of policymaking. Due to the fact that the awareness of policy-makers regarding the public perspectives and viewpoints is of a considerable importance in developing and performing the strategies and programs, engaging individuals in this process is very essential [21]. Given the severity of COVID-19 pandemic and the double need for the cooperation of individuals in the process of controlling this pandemic, understanding the public perspectives would be crucial and instructive. This cross-sectional survey aimed to explore such perspectives towards the current pandemic among the Iranian. Indeed, we sought to provide a favorable platform for effective policies in the face of the COVID-19 pandemic through recognizing the public expectations.

## Methods

### Study design and setting

This cross-sectional survey used an open-ended online questionnaire to investigate the common perspectives and perceptions among the Iranians in the current COVID-19 pandemic and inform the policy-makers about expectations of people from the government. This study was conducted by the Health Policy Research Center, Shiraz University of Medical Sciences (SUMS), Shiraz, Iran, during February to March 2020.

### Sampling and recruitment

In current study, the process of statistical population sampling was carried out using snowball and convenient sampling techniques. In addition, the highest heterogeneity was secured through classifying the individual based on the gender, age, job status, level of education, marital status, and geographical regions. Before mailing the link of the questionnaire, an invitation letter containing detailed information about the study (e.g., objectives of the study and anonymity) was submitted to the participants via social media *(*i.e. WhatsApp, Instagram, etc.). Furthermore, a written informed form along with the questionnaire was sent to the volunteer participants.

### Data collection

A self-designed questionnaire was developed and used in this online survey in order to explore the expectations of citizens from the government during the current COVID-19 pandemic. The questionnaire contained three open-ended questions:

1. “What are the main questions of you or those around you about this new virus?”
2. “What are the main concerns of you or those around you about this new virus?”
3. “What are the main doubts of you or those around you about this new virus?”

The first author (a female PhD in Pharmacoeconomics & Pharma Management), as a health researcher with scientific and executive background in the public health, could facilitate the investigations in this stage. According to a pilot investigation, the questions were revised to improve the clarity. In addition, an invitation letter and a written consent form that included information about research purposes and ethical issues, were provided for the individuals and all participants gave their written consent to participate in the study. In this process, a population-based random sampling of Iranian adults at the age of 18 years or more was carried out through social media with the purpose of questionnaire completion. However, the research team identified one or more focal points in each province to maintain the national status of the study. Therefore, the link was only sent to those participants that were able to answer the questions.

The initial questionnaire was delivered to five academic research experts to validate the clarity, comprehensiveness, and relevance of the questions. During the data collection process, answers were anonymously transcribed and saved in the Windows Microsoft to accelerate the analysis. A blank copy of the questionnaire provides as the supplementary file 1.

### Data analysis

The collected data were analyzed and described using a thematic analysis approach. Three of the authors (LZ, AKS, and STH) participated in the analysis process in order to read and codify the transcribed texts independently and consequently, extract the meaning units. Then, the detected codes were assessed with the purpose of identifying the sub-themes. Finally, the possible connections among the manifested sub-themes were monitored, and the main themes were achieved. During this process, all disagreements between the coders were solved through the discussion and consensus strategies. Furthermore, the authors with different scientific backgrounds and interests participated in the analysis process in order to foster the critical reflexivity and reduce the risk of bias [22]. The analysis was performed manually; moreover, the analyzers used MAXQDA software version 11 if required (VERBI GmbH Berlin, Germany).

### Ethical approval

Research ethics confirmation was granted by the Research Ethics Committee at the Shiraz University of Medical Sciences (IR.SUMS.REC.1399.090).

## Results

Generally, 2547 participants agreed to participate in this study and completed the online questionnaire (Table 1). The Iranian had many expectations from their government regarding its response to COVID-19 pandemic. Because the pandemic had created a kind of despair in the public sphere, the citizens expected serious and effective measures to be adopted by the government. In addition to the personal concerns, they had serious social concerns that were mostly concerned with other problems of citizens resulted from this outbreak. On the other hand, the impacts of this epidemic on losing jobs and conditions of poor people were among the most frequently mentioned concerns, as the participants represented several expectations in line with these concerns. Considering the expectations of people from the government to combat COVID-19 pandemic, three major themes were extracted: (1) Health-related expectations, (2) policy-related expectations, and (3) Information-related expectations. Furthermore, each major theme had several subthemes, as shown in Table 2. In the following sections, the findings of the analysis process along with direct quotations from the answers of participants are described in details.

**Table 1:** Characteristics of participants

Total sample (n)	2547
**Age, Mean (SD)**	36.38 (10.64)
**Male, n (%)**	1246 (48.9)
**Education level, n (%)**	
Under diploma	149 (5.8)
Diploma	311 (12.2)
Associate degree	195 (7.6)
BSc	850 (33.3)
MSc	684 (26.8)
PhD	358 (14.0)
Missing	3 (0.1)
**Marital status, n (%)**	
Single	799 (31.3)
Married	1698 (66.6)
Divorced	34 (1.3)
Wife died	16 (0.6)
Missing	4 (0.2)
**Employment status, n (%)**	
Government employment	760 (29.8)
Non-government employment	365 (14.3)
Self-employment	319 (12.5)
Student	382 (15.0)
Housewife	307 (12.0)
Retired	120 (4.7)
Unemployed (job seeker)	160 (6.3)
Unemployed	15 (0.6)
Day worker	103 (4.0)
Missing	19 (0.7)
**Income level, n (%)**	
Below the poverty line	592 (23.2)
Poverty line	1133 (44.4)
Above the poverty line	816 (32.0)
Missing	9 (0.4)

**Table 2:** Expectations of citizens from the government in response to COVID 19 outbreak in Iran

Main themes	Sub-themes	Codes
**Health-related expectations**	Paying attention to patients and their family members Providing their needs	▪ Following up patients during quarantine ▪ Providing attention and support to patients’ families ▪ Monitoring patients’ families ▪ Providing patients with health and hygiene facilities
	Justice in medical care Providing health support Paying attention to deprived people	▪ Providing equitable medical care ▪ Providing health packages to household ▪ Distributing masks and gloves via stores ▪ Disinfecting passages and public places ▪ Providing health services to deprived regions ▪ Producing and distributing medicines sufficiently
**Political-related expectations**	Monitoring quarantine Appling strict restrictions Closing borders	▪ Compulsory quarantine ▪ Timely quarantine ▪ Cash penalty ▪ Closing intercity roads ▪ Restricting travels ▪ Canceling foreign flights ▪ Closing all public centers ▪ Setting new traffic rules ▪ Dealing with lawbreakers seriously ▪ Dealing with rumor mongers seriously
	Governance trust Use the experiences of other countries Appling serious monitoring and control Foreign cooperation	▪ Officials’ honesty ▪ Crisis management by experts ▪ Using the experiences of other countries in pandemic control ▪ Providing healthcare centers with necessary permissions ▪ Fair distribution of medical services and equipment ▪ Monitoring the production and distribution of bread ▪ Cooperation and coordination of government agencies
	Providing financial support Livelihood assistance	▪ Deferment of bank installments ▪ Non-payment of service bills ▪ Providing livelihood assistance packages ▪ Giving loans at low interest rates to some businesses
**Information-related expectations**	Adopting honesty Statistics transparency	▪ Providing accurate news and statistics ▪ Providing transparency on transmission routes ▪ Providing transparency on the prevalence rate ▪ Providing transparency on high-risk groups ▪ Preventing the spread of false news ▪ Announcing the exact statistics of those affected by city
	Citizen education Health literacy Comprehensive and inclusive health education	▪ Providing appropriate preventive training ▪ Teaching healthy behaviors ▪ Teaching how to use e-services ▪ Providing life-style change training ▪ Providing the required diet and exercise programs

### Health-related expectations

In this study, a majority of the participants highlighted the need to consider some important factors for the infected patients and their families with the purpose of preventing further transmission. Breaking the transmission chain as well as the provision of financial and hygiene support during the pandemic was another main expectation. In other words, they believed if the required support was not provided to the infected patients, there would still be the possibility of virus transmission. In addition, the fair and equitable provision of medical and preventive services to the public, especially the vulnerable groups, was the other demand of participants. As a remarkable finding, the participants described the need for continuous monitoring and screening of relatives and families of t he patients in order to interrupt the transmission chain.

*"If the patient is not monitored continuously during the quarantine, the virus may be transmitted to the other individuals."*

*"Households, especially those with patients, should be provided with financial supports to prevent them from attending outside. On the other hand, more sanitary facilities should be provided to these groups."*

*"Unfortunately, in some cities, the relatives and families of those infected with coronavirus are not concerned as the potential carriers of this virus. I think health centers should monitor them constantly."*

Providing sufficient health supports was another subtheme, which contained a number of codes such as equitable medical care services. Many participants pointed to the unfair access to health care services in Iran and the importance of resolving this problem to facilitate the fight against COVID-19 outbreak. Moreover, some other participants believed that health packages should be submitted to households by the government and local institutions because many of people cannot afford to buy the necessary personal protective equipment due to the economic crisis. A number of these individuals also pointed to the poor distribution of preventive measures such as masks and gloves across the country, which led to increased prices, corruption, and difficult access. Accordingly, they proposed the formation of a codified and transparent distribution structure by the government. For example, they referred to various solutions such as using the capacities of the local stores to distribute the aforementioned items and goods. Additionally, the importance of disinfecting the public passages and places were also noted. Briefly, the participants stated that rationale and continuous disinfection was vital according to the findings of researchers regarding the rapid spread of the virus in public places. More importantly, paying attention to vulnerable groups and responding to their needs appropriately was another common expectation in this study.

*"Direct payment for health services is very high in Iran. Although the government stated that costs of treating the coronavirus patients were to be covered by the insurances, there are still individuals who have confronted with many challenges regarding the transportation and accommodation costs."*

*"It is constantly recommended that people wear a mask, but there is no mask! Maybe there is, but not available to us. The government must create a proper structure for the distribution of these devices."*

*"Experts have recently stated that the virus would stay in the air for a longer period. I think the crowded centers should be more disinfected. Although much effort is being made now, they have to be more effective."*

### Political-related expectations

Our findings indicated various expectations regarding the political issues. The imposition of strict restrictions, adopting serious monitoring and controlling procedures, and providing financial support were recognized as the most important factors. With regard to the adoption of quarantine policies in some countries, the participants called for more serious policies on compulsory quarantine. On the other hand, some of them believed that the government had not implemented quarantine policies in a timely manner, and that it would be better if they took actions as soon as possible. According to another finding, the effective punitive measures have to be considered for those who do not follow the containment measures. In current study, some of the participants referred to the likelihood for the disease entering from other countries and asked the government to cancel all foreign flights. Since widespread rumors and misinformation are common after crises, serious measures against the rumor mongers would be a potential solution.

*"Many people do not pay attention to quarantine and travel. The government must respond this very seriously. I think it should be a mandatory quarantine, like in European countries."*

*"Iran started quarantine too late. Home quarantine must have been started since the first day so that the disease would not have spread to other cities."*

*"There are a number of punishments, but they are not enough. Most of these punishments are limited to cars and road transportation. I think individual fines should also be considered for individuals."*

Many participants expected the authorities to be honest and transparent in providing reports and advice. They argued that honesty could be a proper incentive for citizens to participate and cooperate with the made decisions. Using related experts and experiences from other countries in managing the crisis was the other expectation noted by some individuals. In addition, the participants, regardless of their position, criticized the management of medical centers and believed that the local centers had no sufficient authority and autonomy to make quick decisions. Thus, delegating authority to local units might facilitate the process of meeting demands during this pandemic. More importantly, a large proportion of the participants identified the fair distribution of medical supplies and equipment, and inter-sectorial cooperation among stakeholders as prerequisites of an effective response to COVID-19 outbreak.

*"Many people have doubts in the sincerity of the authorities. If officials speak more honestly, people will be more supportive."*

*"We must use the experiences of successful countries such as South Korea and even Germany, which have very low mortality rates."*

*"Crisis management is a purely scientific and specialized matter that could be achieved by experienced managers. Experts of this field should be involved as well."*

*"Cooperation and coordination among responsible institutions, and integrated stewardship can be helpful."*

In accordance with the adverse economic effects of pandemics such as COVID-19, there were some recommendations to provide financial supports for the public. The participants noted that bank installments and maturities should be postponed due to the prohibition of commercial activities and a sharp decline in income. Moreover, the non-payment of service bills (water, energy, etc.) and the provision of livelihood packages by the government were also expected by the participants. Supplying the low-interest loans to enhance the liquidity for commercial units and the general public during this recession was also another expectation.

*"Although we have no income, the bank has withdrawn the installments. In this situation, banks should be more considerate."*

*" … especially for poor households, the payment of service bills must be canceled or at least postponed."*

*"In this recession, it is possible to facilitate the required liquidity by providing low-interest loans."*

### Information-related expectations

In this study, the participants expressed that adopting honesty and transparency principals by news agencies would promote the social capital. In addition, accurate news and statistics would prevent people from visiting invalid and fake news sources. Many participants believed that there was a need for more comprehensive information about the SARS-CoV-2 transmissions to prevent obsessions and other potential psychological disorders. Finding out how vulnerable groups are affected by the pandemic as well as its side effects were another point stated by the study participants. Remarkably, they believed that announcing the related information and statistics could be effective in informing citizens and adhering to the proposed principles.

*"The official media must represent the real news to make people more confident."*

*"If the real news and information is provided, no one will refer to the rumors and fake news."*

*"Elderly people are very worried when it is announced in the news that the disease is more dangerous for this group. More information should be provided on the effects of the disease on the elderly and other high-risk groups."*

Finally, the provision of comprehensive educational programs was another expectation of participants from policy-makers in response to COVID-19 pandemic. According to the findings, proper training in terms of the virus transmission prevention methods, healthy behaviors, and how to use e-services were the common expectations. Generally, many believed that the general public was not fully aware of the correct preventive methods, and the capacity of the official and virtual media could be further exploited. One of the interesting findings was the role of instruction in the process of changing the lifestyles based on conditions resulted from this pandemic. Due to the fact that the implementation of quarantine policies may affect the nutritional and physical conditions of individuals, the provision of accurate educational services would prevent many complications.

*"The media should provide relevant educational programs tailored to the perceptions of different people in the community. Many programs use specialized terms that are not understandable to many people."*

*"Many individuals are not aware of e-services. Relevant training programs can be provided to prevent their direct presence in banks and organizations."*

*"I am very worried about my children's eating habits and physical condition during this period. I wish some educational programs were provided so that we could act based on them."*

## Discussion

This cross-sectional study aimed to explore the expectations of the Iranian from the government during COVID-19 pandemic. The detected themes indicated that the citizens had high expectations from their government. It was noticed that people had different expectations regarding responses of the government to this pandemic, which encompassed health-related, policy-related, and Information-related expectations.

Our findings point to the importance of optimal patient quarantine during boredom and after discharge since the virus is transmitted quickly. In addition, the general public expects support the submission of health and financial services to families with the affected patients in order to make them able to tackle with the disease more appropriately. In this regard, a number of countries such as China, France, and Italy implemented highly strict quarantine policies after confirming the first positive cases [23–25]. However, as Iran was tackling with several economic and social problems at the same time [7, 26], it initially focused more on advisory policies and public closures and announced tougher quarantine policies such as prohibiting intercity transportation and shopping malls a few weeks later [5]. In this case, many participants criticized the delay of the government. According to this finding, a rapid review by Nussbaumer-Streit et al. demonstrated that early quarantine had better effects [27]. Given the importance of breaking the virus transmission chain, many participants mentioned that the continuous monitoring of relatives and families of infected patients is highly important. Although Iran’s Ministry of Health is working on a national screening plan to identify and follow the suspicious cases before the symptoms appear [5], the infected people of some cities come to public places after being discharged and getting recovered, and this is an issue requiring further preventive policies. Accordingly, South Korea, which is considered as one of the most successful countries in the fight against the disease, has adopted a policy to track the corona virus carriers based on which people that are diagnosed with the disease would have an app installed on their mobile phone [28]. In this regard, without disclosing the personal information of infected people, their presence in different places would be notified to other members of the community [29].

The provision of equitable and affordable health care services was another expectation. Indeed, economic hardships are one of the most important causes of stress, as shown in a study carried out in Bangladesh [30]. Regarding the high cost of health care services in Iran, many people face with financial hardships [31]. In response, the government of Iran has developed a number of policies to increase the access to health care services for COVID-19 patients. Therefore, the services are completely free for the groups covered by the health insurance, and there are also some exemptions for the same group [32]. It should also be noted that Iran has provided free health services to immigrants during this pandemic [33]. Continuous disinfection of public places is another expectation to prevent the virus transmission. As evidence shows, almost all the affected countries disinfect public places and routes [34]. Our findings indicate that despite efforts to disinfect public places, many individuals do not consider them to be sufficient and believe that they should be increased in quality and quantity. Although wearing masks and gloves has been emphasized to prevent the infection [6, 26], it is still difficult to have access to these protective devices in Iran; therefore, more appropriate production and distribution mechanisms must be adopted. However, the shortage of such equipment after the crisis is not unexpected, as observed in developed countries such as the U.S. and Italy [35, 36].

COVID-19 pandemic has been considered as a global political crisis [37]. In Iran, there have been a number of political problems posed by this pandemic. According to the findings, stricter enforcement measures during the quarantine period and the imposition of effective fines were expected by the participants. From another perspective, many participants stated that as it was applied in Wuhan, traveling from cities such as Qom and Tehran, which were the sources of the outbreak, should be prohibited [25]. However, this strategy could not been practically applied due to the limitations existed for the government of Iran resulted from international sanctions [7]. Many participants also referred to serious actions against lawbreakers and rumor mongers as an effective approach in facilitating the fight against COVID-19. Evidence suggests that countries such as India and France have imposed severe penalties on lawbreakers with the purpose of slowing down the spread of the virus [38, 39]. Saudi Arabia, also imposed heavy fines on violators during COVID-19 outbreak based on its experiences with MERS pandemic [40].

Honesty and transparency are the best policies in the crisis management [41]. As evidence indicates, openness and honesty can promote the fight against the pandemic, as observed in the case of SARS in 2003 in Hong Kong and Taiwan [42, 43]. Moreover, the participants emphasized the importance of the honesty of authorities and expressed it as a requirement for public trust. Findings indicated the need for greater use of experts in COVID-19 control process in Iran. On the other hand, since coordination and collaboration among stakeholders and actors is a crucial prerequisite for effective policy-making and implementation of programs and strategies [44], a majority of the participants highlighted this point. Also, the need to delegate authority to relevant local institutions and centers with the purpose of expediting the decision-making process was another finding of this study. Unfortunately, the existence of slow bureaucratic structures in Iran has always been considered as one of the main obstacles in the face of crises [45]. Notably, Norway was one of the most successful countries in controlling the COVID-19 using the capacity of regional and local enterprises such as municipalities [46]. It is noteworthy that due to the delegation of strong power to local organizations in Japan, local governments in Tokyo and Hokkaido responded to the COVID-19 outbreak earlier than the national government [47].

The negative economic effects of pandemics have always been a major challenge to societies [48, 49]. During a short period after the COVID-19 outbreak, the world economy faced many problems; therefore, the citizens, especially the vulnerable, have a range of expectations from their governments [50]. According to our findings and economic problems, the participants declared that delaying the bank installments, providing low-interest loans, submitting livelihood packages to poor households, and considering exemptions from non-payment of service bills could be potential policies. Different countries have pursued various financial policies in facing with the disease. For example, South Korea allocates approximately $1030 per month on four-member families with an infected patient [51]. Similar policies have been also adopted by Italy, the United States, Germany, Japan and France [52]. Furthermore, the British government has set aside about $400 billion to support small and large businesses in the form of guaranteed loans [52]. In addition, the Swiss central bank called on its banks to refrain from paying dividends and repurchasing stocks as the government seeks to increase funding in order to prevent the industry stagnation. The Swedish Financial Supervisory Authority has also announced that it has allowed banks to defer the re-payment of mortgage installments [53]. Since the International Monetary Fund has forecasted a negative 3% global growth in 2020 [54], various countries, including Iran, need to develop comprehensive policies to reduce the negative economic effects of COVID-19 pandemic.

Experts believe that the main role of media in different crises, such as pandemics, is to publish the right news, reduce the pessimism towards the information and news of official sources, and create peace of mind [55–57]. In current study, participants demanded for the provision of real time, and accurate news and statistics by the official media; also, and many believed that the distrust of people had turned them to unofficial sources. In addition, mass media play a significant role during pandemics such as effective health communication for consideration of the preventive measures, appropriate approaches of helping individuals to deal with the social distancing, reduction of inequalities, stigma, and psychological disorders [58]. In other words, media are acknowledged as a main tool in risk communication during this situation; however, the impacts of media on the pandemics are complex [59]. Briefly, considering the importance of information and their potential benefits can be an appropriate solution to reduce the adverse effects of COVID-19 epidemic.

### Limitations

Two of the most important limitations of the current study should be mentioned before generalizing the findings. First, current study did not conduct the conventional qualitative methods, i.e., interviews or discussion, and the data collected through textual information online. Second, it is a cross-sectional study so that we can not make any transformations in perspectives or perceptions based on changes in the COVID-19 epidemic.

## Conclusions

Generally, three main themes were recognized as the main expectations from government to fight COVID-19 pandemic including health-related expectations, policy-related expectations, and Information-related expectations. Results showed that people would face a number of challenges during this pandemic; therefore, they have to expect the government and other responsible institutions to minimize their burden by adopting effective policies. Furthermore, the findings of this study could help the policy-makers become more aware of the expectations of people and develop better strategies. It is suggested that governments be aware of the requirements of their citizens during the epidemics and adjust their policies accordingly. Quantitative studies in this field are also recommended.

## Supplementary Information

**Additional file 1.** The questionnaire: A blank copy of the questionnaire.

## Data Availability

The data collected and analyzed during the study are available from the corresponding author on reasonable request.

## Abbreviations

SARS-CoV-2: Severe Acute Respiratory Syndrome Coronavirus 2
IRB: Institution Review Board
UN: United Nations
SUMS: Shiraz University of Medical Sciences
SARS: Severe Acute Respiratory Syndrome
WHO: World Health Organization
