# Supplementary material for: Expectations of citizens from the government in response to COVID-19 pandemic: a cross-sectional study in Iran
Source: BMC Public Health. 2021 Apr 8;21:686. doi: 10.1186/s12889-021-10722-y (PMC8027969; doi:10.1186/s12889-021-10722-y)
Supplement: Supplementary file 1 — Additional file 1. The questionnaire: A blank copy of the questionnaire. [file 12889_2021_10722_MOESM1_ESM.docx]

**The esteemed participant,**

My name is …. The present questionnaire aims to contribute to the existing knowledge on what citizens’ expectations from government in response to COVID-19 Pandemic. It takes approximately 10 minutes to complete this questionnaire. Your participation in this study is entirely voluntary. The information and data are strictly confidential. We will publish only aggregated, anonymous results.

Thank you for your support!

**Answer Guide:**

Please, choose the most suitable option in your opinion.

Table Q.1: Demographic profile.

| **Age (years):** |
| --- |
| **Gender:** |
| **Marital status:** |
| **Employment status:** |
| **Education:** |
| **Income level:**   - Below the poverty line - Poverty line - Above the poverty line |
| **Tel/ Phone (Optional):** |

Table Q.2: The questions.

1. What are the main questions of you or those around you about this new virus?"
2. "What are the main concerns of you or those around you about this new virus?"
3. "What are the main doubts of you or those around you about this new virus?"

Thank you very much for your participation.
